# Acute myeloid leukaemia: challenges and real world data from India

**DOI:** 10.1111/bjh.13406

**Published:** 2015-04-09

**Authors:** Chepsy Philip, Biju George, Abhijeet Ganapule, Anu Korula, Punit Jain, Ansu Abu Alex, Kavitha M. Lakshmi, Usha Sitaram, Fouzia N. Abubacker, Aby Abraham, Auro Viswabandya, Vivi M. Srivastava, Alok Srivastava, Poonkuzhali Balasubramanian, Vikram Mathews

**Affiliations:** ^1^ Department of HaematologyChristian Medical CollegeVelloreIndia; ^2^ Department of Transfusion Medicine and ImmunohaematologyChristian Medical CollegeVelloreIndia; ^3^ Cytogenetics UnitChristian Medical CollegeVelloreIndia

**Keywords:** acute myeloid leukaemia, real world data, cost of therapy, induction mortality, multidrug‐resistant bacteria

## Abstract

The management of acute myeloid leukaemia (AML) in India remains a challenge. In a two‐year prospective study at our centre there were 380 newly diagnosed AML (excluding acute promyelocytic leukaemia, AML‐M3) patients. The median age of newly diagnosed patients was 40 years (range: 1–79; 12·3% were ≤ 15 years, 16·3% were ≥ 60 years old) and there were 244 (64·2%) males. The median duration of symptoms prior to first presentation at our hospital was 4 weeks (range: 1–52). The median distance from home to hospital was 580 km (range: 6–3200 km). 109 (29%) opted for standard of care and were admitted for induction chemotherapy. Of the 271 that did not take treatment the major reason was lack of financial resources in 219 (81%). There were 27 (24·7%) inductions deaths and of these, 12 (44·5%) were due to multidrug‐resistant gram‐negative bacilli and 12 (44·5%) showed evidence of a fungal infection. The overall survival at 1 year was 70·4% ± 10·7%, 55·6% ± 6·8% and 42·4% ± 15·6% in patients aged ≤15 years, 15 ‐ 60 years and ≥60 years, respectively. In conclusion, the biggest constraint is the cost of treatment and the absence of a health security net to treat all patients with this diagnosis.

It is well recognized that there are significant financial challenges in treating acute myeloid leukaemia (AML) (Redaelli *et al*, 2004). There is an acknowledged paucity of data to address the issue of the economic burden of AML, even in high‐income countries with well‐established and well‐monitored health care delivery systems (Uyl‐de Groot *et al*, 2001; Redaelli *et al*, 2004; Leunis *et al*, 2012). From the available data, the cost of treating AML in a developed country varies from US$ 80 000 to >150 000/patient (Stalfelt & Brodin, 1994; Uyl‐de Groot *et al*, 2001; Redaelli *et al*, 2004; Leunis *et al*, 2012). These figures have to be put in context with the fact that 70% of the countries in the world, which contribute to >75% of the world's population, have a gross national income (GNI)/capita of less than US$ 10 000, Wikipedia (2014) and in many of these countries the cost of medical care is met by self‐payment. Registry‐based data on patients actually diagnosed, receiving treatment and their clinical outcomes have significant limitations with the recognition that, in often quoted registry data, such as that of the Surveillance Epidemiology and End Results (SEER)‐Medicare database, there is up to 50% under reporting of the diagnosis of AML (Craig *et al*, 2012). Significant advances have been made in the management of AML. However, majority of these advances are based on well controlled clinical trials from countries with universal health care access and have a significant bias in the patients enrolled being younger, having better performance scores and less co‐morbidities than the average patient with a diagnosis of AML. The universal applicability or relevance of many of these advances, which often involve more intensive chemotherapy and stem cell transplantation, have rarely been addressed. There is very limited real world data of the clinical outcome of patients with a diagnosis of AML where the denominator includes every patient diagnosed with AML. When such data is available, the observations and conclusions often seem at odds with published clinical trial data (Juliusson *et al*, 2012).

While India has access to highly skilled medical professionals and institutes that can provide state of the art medical care comparable to the best in the world, these are often limited to major cities and are inaccessible to the poor. The overall number of trained educated health workers is very low in proportion to the country's population and the effect of this is reflected in the dismal population health indices (Horton & Das, 2011). India is ranked 135 in the world in terms of the human development index (HDI), which assesses the long‐term progress in health and social well‐being of a country (Malik, 2014). Only 1·2% of the country's GDP goes towards health and an estimated 55% of the population in India is poor by the multidimensional poverty index (MPI) (Horton & Das, 2011). It is estimated that 80% of medical expenses is met by out of pocket expenses, resulting in approximately 39 million people falling below the poverty line every year (Garg & Karan, 2009; Balarajan *et al*, 2011). It is widely recognized that there is significant under diagnosis and under reporting of AML in India and that a substantial proportion of patients do not receive standard of care therapy. However, these issues have never been systematically evaluated. The objective of this descriptive study was to document the number of newly diagnosed with AML over a fixed period of time, the proportion that did not proceed with standard therapy and the clinical outcome of those receiving standard of care, and to analyse the potential challenges in each of these areas.

## Patients and methods

This prospective and descriptive single centre study was approved by the Institutional research and ethics review board. All patients with a diagnosis of AML (excluding AML‐M3 [acute promyelocytic leukaemia]) who presented to the Department of Clinical Haematology at our institution over a two‐year period, from 1 July 2012 to 30 June 2014, were enrolled after obtaining written and informed consent. Demographic details were recorded in a questionnaire format at first contact (Appendix S1).

## Diagnosis

A diagnosis of AML by French‐American‐British (FAB) criteria (Bennett *et al*, 1976) was sufficient for enrolment in the study. However, for those patients who opted to proceed with definitive therapy a more detailed evaluation was undertaken and the diagnosis of AML and subtype was made as per the World Health Organization (WHO) classification (Swerdlow, 2008). This included morphological assessment of the bone marrow aspiration and biopsy, immunophenotyping, karyotyping and molecular screening for mutations in the *NPM1* and *FLT3* genes, as previously reported (Yamamoto *et al*, 2001; Chendamarai *et al*, 2012; Parihar *et al*, 2012).

## Treatment plan

Patients choosing to proceed with treatment were admitted to the inpatient wards. Paediatric patients (≤15 years) were treated with the Berlin‐Frankfürt‐Münster (BFM) AML‐BFM98 protocol (Creutzig *et al*, 2006) and were offered an allogeneic stem cell transplantation (SCT) in first remission (CR1) only if they were in the high risk group. Young adults (>15 – <60 years) received induction chemotherapy with daunorubicin 60 mg/m^2^/d × 3 days along with cytosine arabinoside 200 mg/m^2^/d as a continuous infusion × 7 days (7/3). Complete remission was documented as per standardized reporting criteria (Cheson *et al*, 2003). In young adults, good risk patients were only offered 3 cycles of high dose cytosine arabinoside consolidation (HiDAC; 3 g/m^2^/dose q12 h on days 1, 3 and 5/cycle); intermediate‐ and high‐risk patients were offered an allogeneic SCT in CR1. Elderly patients (≥60 years) and young adults with poor performance status and multiple co‐morbidities received hypomethylating agents (azacitidine or decitabine) at the discretion of the treating physician (Fenaux *et al*, 2009; Cashen *et al*, 2010).

## Supportive care

Induction and consolidation chemotherapy was administered to all patients in an inpatient ward without specialized air handling facilities. Post‐induction patients remained in the inpatient facility until they had recovered from neutropenia and were clinically stable. If clinically stable, patients were discharged post‐chemotherapy consolidation and re‐admitted if they developed febrile neutropenia or any other complication that the primary physician felt warranted admission and close observation. Post‐allogeneic SCT patients were usually discharged when there was evidence of stable engraftment. Following initial discharge, patients who underwent an allogeneic SCT remained close to the hospital for a minimum period of 2 to 3 months, longer if they developed significant graft‐versus‐host disease. All patients received prophylactic antifungal therapy with either posaconazole or amphotericin. Antibiotic policy for febrile neutropenia followed the principles laid out by the Infectious Disease Society of America (IDSA) (Freifeld *et al*, 2011). Blood cultures were obtained prior to both starting and escalation of antibiotic therapy. Additional blood cultures were obtained as required for persistent fever. When blood culture identified an organism the antibiotics were changed based on the antibiogram. If the clinical condition of the patient deteriorated such that they required blood pressure (inotropic) or ventilatory support, the patient was moved to an intensive care unit. The trigger for platelet transfusion was a platelet count of <10 × 10^9^/l in the absence of bleeding and <50 × 10^9^/l if there was active bleeding. The trigger for a red blood cell transfusion was a haemoglobin level <80 g/l. Granulocyte colony‐stimulating factor was administered in the induction and consolidation phases when neutropenia was established and this was continued until the absolute neutrophil count was > 1 × 10^9^/l.

## Management of patients not opting for treatment at our centre

Those who chose not to proceed with treatment were discharged after stabilization (when required). Patients were usually discharged with a suggested plan of management in case they opted to take treatment at another centre. Follow‐up telephone interviews were made periodically until July 2014. In those who were deceased, the information was collected from a relative over the telephone. We were not able to establish telephonic contact with any of the patients who resided outside the country.

## Definitions

Standard reporting practices were used for definition of remission, relapse and leukaemia‐free state (Cheson *et al*, 2003). The cytogenetic risk groups were clasified based on the refined UK Medical Research Council criteria into favourable, intermediate and adverse risk groups (Grimwade *et al*, 2010). Invasive fungal infection (IFI) categories of possible, probable and proven IFI were structured on definitions from the European Organization for Research and Treatment of Cancer/Invasive Fungal Infections Cooperative Group and the National Institute of Allergy and Infectious Diseases Mycoses Study Group (EORTC/MSG) Consensus Group (De Pauw *et al*, 2008). Carbapenem‐resistant enterobacteriaceae (CRE) infections were identified based on the US Centers for Disease Control interim surveillance definitions as enterobacteriaceae with non‐susceptibility to carbapenem and resistance to third generation cephalosporins (Kallen & Guh, 2012).

## Statistics

Descriptive statistics were calculated for all variables. Differences in proportions were assessed using the χ2 or Fisher exact statistic. Differences in means were tested using a Mann–Whitney‐*U* test or t‐test as appropriate. The probability of survival was estimated with the use of the product‐limit method of Kaplan and Meier and compared by the log‐rank test. All survival estimates were reported ± 1 standard error (SE). All *P* values were 2‐sided, with values ≤0·05 indicating statistical significance. Statistical analysis was performed using spss 16.0 software (SPSS, Chicago, IL, USA).

## Results

### Patient accrual and baseline characteristics

**Figure 1:**
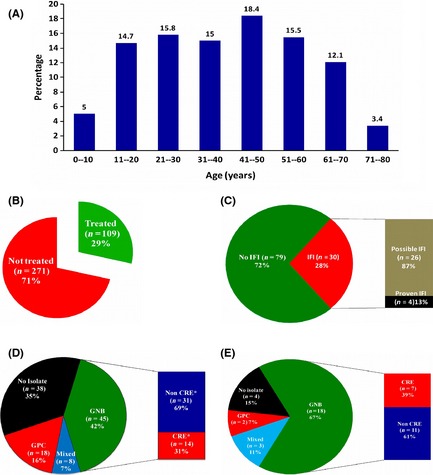
(A) Age distribution of newly diagnosed cases of acute myeloid leukaemia. (B) Proportion of newly diagnosed patients that opted for treatment (*n* = 109). (C) For the 109 patients that opted for treatment at our centre, the proportion of patients that had evidence of invasive fungal disease (IFI) by the revised EORTC/MSG criteria (De Pauw *et al*, 2008). (D) The pattern of documented bacterial infections for the 109 patients that received induction therapy. Data mutually exclusive for each patient, i.e., if multiple cultures were positive for one or more gram‐negative bacilli (GNB), data counted only once as GNB, similarly for gram‐positive cocci (GPC) and documented here as mixed if both GNB and GPC were identified in the same or different cultures of one patient. CRE: carbapenem‐resistant enterobacteriaceae (Kallen & Guh, 2012) E) The pattern of bacterial infections for the 27 patients that died during induction [see above (panel 1D) for description of data].

Over the period of this study there were 380 newly diagnosed cases of AML, of which 47 (12·3%) were ≤15 years old, 271 (71·3%) were aged between 15 and <60 years and 62 (16·3%) were ≥ 60 years old. The median age of newly diagnosed patients was 40 years (range: 1–79), the age distribution of newly diagnosed cases is illustrated in Fig 1A. There were 244 (64·2%) males. The median duration of symptoms prior to first presentation at our hospital was 4 weeks (range: 1–52; distribution illustrated in Fig S1). ECOG performance score at presentation was ≥2 in 23%. The median distance from home to hospital was 580 km (range: 6–3200) and 27 patients were from another country (majority from neighbouring countries, such as Bangladesh and Sri Lanka). The baseline demographic characteristics of newly diagnosed patients with AML are summarized in Table 1 and Table S1.

**Table 1:** Baseline demographic characteristics in newly diagnosed patients with AML

Variable	Patients (*n* = 380)*n* (%)/Median (Range)/Mean (±SD)
Age (years)	40 (1–79)
Sex (male)	244 (64·2)
Symptom duration (weeks)	4 (1–52)
Distance from hospital (kilometres)	580 (6–3200)
Proceeded to receive treatment	109 (28·7)
ECOG PS at diagnosis	*n* = 373
0/1	289 (77·5)
2	71 (19)
3/4	13 (3·5)
Haemoglobin (g/l)	77 ± 23·4
White blood cell count (×10^9^/l)	16·3 (0·2–920)
Platelet count (×10^9^/l)	36 (2–394)
Cytogenetic Risk Group	*n* = 247
Favourable	29 (11·8)
t (8;21)	24
inv (16); t (16;16)	5
Intermediate	173 (70)
Adverse	45 (18·2)
monosomy 7	12
del 5q; t(6;9); t(9;22) (1 each)	3
inv 3	2
Complex karyotype	28

ECOG PS, Eastern Cooperative Oncology Group performance score; SD, standard deviation.

### Cytogenetic and molecular data

Cytogenetic data was available in 247 and of these, 29 (11·8%), 173 (70%) and 45 (18·2%) were in the favourable, intermediate and adverse risk groups respectively (Table 1). *FLT3* and *NPM1* mutation status was available in 143 and of these 22 (15·4%), 5 (3·5%) and 17(12%) were *FLT3*‐/*NPM1 *+* *,* FLT3 *+* */*NPM1*‐ and *FLT3 *+* */*NPM1 *+* * respectively (Table S1). Among the 109 patients that proceeded to treatment, an adverse risk cytogenetic finding was noted in 22 (20%) and a *FLT3* mutation was seen in 17 (18·1%).

### Treatment details

Of the 380 newly diagnosed patients, only 109 (29%) opted to have standard of care therapy at our centre (Fig 1B). Of the 109 who received treatment, 23 (21%) were aged ≤15 years (paediatric), 75 (69%) were young adults (15 – <60 years) and 11 (10%) were elderly (≥60 years). All paediatric patients received the AML‐BFM98 protocol (Creutzig *et al*, 2006). Among the young adults, 68 (91%) received a standard induction while the remaining 7 (9%) received a hypomethylating agent as initial induction therapy. Of the 11 elderly patients in this series, 6 (55%) received a hypomethylating agent for induction, 4 (36%) received a standard induction regimen and one patient received an abbreviated induction with 5 days of cytosine arabinoside and 2 days of anthracycline.

Post‐induction, paediatric patients proceeded to receive consolidation and maintenance therapy as per the BFM‐AML98 protocol, 4 (17·4%) paediatric patients received an allogeneic SCT as part of their consolidation. Among the young adults, 18 received an allogeneic SCT and two an autologous SCT as part of consolidation while the rest (*n* = 36) received chemotherapy alone as consolidation. In the elderly subset, one patient received an autologous SCT as part of consolidation while the rest received chemotherapy alone or multiple cycles of hypomethylating agents.

All patients developed febrile neutropenia post‐initiation of induction therapy. During induction therapy, 30 (28%) had evidence of possible or proven IFI (Fig 1C). Bacteria were isolated on at least one blood culture in 71 (65%) patients. In 45 (42%) patients only a gram‐negative bacilli (GNB) was isolated, in 18 (16%) only a gram‐positive cocci (GPC) and in 8 (7%) a mixture of GNB and GPC (data mutually exclusive for patients, multiple cultures being positive for one patient counted only once). Of the GNB that were isolated, a CRE was identified on at least one blood culture in 14 (31%) patients (Fig 1D).

### Induction deaths

Of the 109 patients in whom induction chemotherapy was initiated there were 27 (24·7%) induction deaths. The induction mortality rate was 4/23 (17%) among the paediatric population, 19/75 (25%) in the young adults and 4/11 (36%) in the elderly. The major cause of death was bacterial sepsis and fungal infections. Among the patients who died in induction, bacteria were isolated on at least one blood culture in 23 (85%) patients. Of these patients, only a GNB was isolated in 18 (67%), 2 (7%) had a GPC alone and 3 (11%) had a mixed culture (Fig 1E). Among the 18 patients in whom a GNB was isolated, the bacterium was a CRE on at least one blood culture in 7 (39%) cases (Fig 1E). Among the patients in whom a GNB was isolated but were not CRE, 5 had growth of a carbapenem‐resistant pseudomonas and hence 12 (44·5%) of the induction deaths could be attributed to MDR GNB. Twelve (44%) of the patients who died in induction had evidence of a possible or definitive IFI. The median time to induction death was 24 days (range: 15 – 63).

### Survival

**Figure 2:**
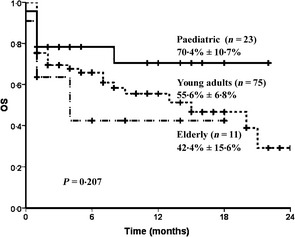
Overall survival in the paediatric population (age ≤15 years), in young adults (age >15 – <60 years) and the elderly (>60 years).

An additional 17 patients died post‐induction during the period of this study. Of these, the majority (*n* = 9) of deaths was due to disease recurrence and eight patients died in remission during subsequent neutropenia of consolidation (6 related to infections and two early post‐transplant treatment‐related mortality). At a median follow‐up of 7 months the overall survival at 1 year was 70·4% ± 10·7%, 55·6% ± 6·8% and 42·4% ± 15·6% in patients aged ≤ 15 years, 15 to <60 years and ≥ 60 years, respectively (Fig 2). Overall survival analysis is limited by the small number of patients in each arm and the short duration of follow‐up in this study.

### Patients not receiving treatment

Two hundred and seventy‐one (71%) of the 380 newly diagnosed patients over the period of this study opted not to have treatment at our centre. The baseline characteristics of these 271 patients were compared with those of patients that went on to receive treatment at our centre (summarized in Table S1). The patients who opted not to have treatment at our centre were significantly older, lived further away from the hospital and had a significantly longer duration of symptoms prior to presenting to our centre. Of the 271 patients who did not receive treatment, the data on reasons for not proceeding with treatment were available in 261 and are summarized in Table 2. The major cause for not proceeding with treatment was lack of financial resources (81%) followed by lack of social support during the period of hospitalization. On follow‐up telephone interviews (*n* = 146) it was noted that 32/146 (22%) of these patients received standard of care therapy at another centre including two who received an allogeneic SCT. Of these, 16 remained alive and 16 had died during the study period and following the last telephone interview. Of the 114/146 (78%) who had not received definitive therapy for AML, 25 were alive at last telephonic follow‐up, all of whom had been diagnosed in the last 6 months of the study. The Kaplan–Meir estimate of median survival in this group was 1 month (95% confidence interval: 0·89 – 1·10).

**Table 2:** Summary of reasons for not proceeding with treatment

Variable^a^	Patients (*n* = 271)*n* (%)
Lack of financial resources to proceed with treatment	219 (81)
Alternative medicine (Ayurveda/Homeopathy/Traditional/Native)	5 (1·9)
Lack of social support	45 (16·6)
Concerned about toxicity of chemotherapy	26 (9·5)
Apathy and fatalistic attitude	17 (6·2)
Preferred to seek treatment elsewhere	4 (1·4)
Data not available	10 (3·7)

a More than one reason allowed per patient.

### Financial burden of therapy

The number of elderly patients was too few and treatment received too varied to make any conclusion and were hence not included in the analysis. The cost of induction, consolidation and allogeneic SCT is summarized in Table 3. The major cost of chemotherapy was during the initial induction therapy followed by an allogeneic SCT. Patients who died during induction and those that received a hypomethylating agent as part of induction or consolidation were excluded from the analysis.

**Table 3:** Cost of therapy

Phase of treatment	≤15 years	>15–<60 years
Induction	*n* = 19^a^	*n* = 49^b^
Mean	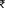 523 543 ($8500)	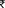 742 387 ($12 300)
Standard deviation	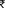 176 800 ($2900)	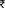 386 991 ($ 6 344)
Consolidation	*n* = 14^@^	*n* = 25^#^
Mean	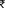 1 231 965 ($20 200)	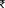 318 128 ($5200)
Standard deviation	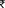 695 546 ($11 400)	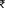 135 040 ($2200)
Transplant	*n* = 4^^^	*n* = 14^^^
Mean	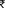 1 462 586 ($23 574)	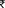 1 983 051 ($32 500)
Standard deviation	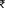 588 099 ($9600)	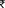 1 031 835 ($16 900)

a Excludes induction deaths.

b Excludes induction deaths and those that received hypomethylating agents.

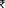
: Indian Rupee :United Statesdollar (calculationbasedonone US = 61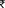). @ Includes 4 cycles of consolidation chemotherapy as part of AML‐BFM 98 protocol. # Only for those that received high dose cytosine arabinoside (HiDAC) consolidation (25 patients and 56 consolidations); the calculated amount is cost per cycle of HiDAC consolidation. ^ Allogeneic stem cell transplantation only. Calculated cost includes all costs incurred over multiple admissions over the period of this study.

## Discussion

This single centre prospective study over a two‐year period illustrates the challenges faced in treating AML in India. Different treatment centres in India are likely to have different costing structures, based on whether they are private for‐profit, private non‐profit and fully or partly government subsidized hospitals. Similarly, there is also likely to be significant heterogeneity between different hospitals with regards to diagnostic facilities available, allogeneic SCT facility and access to trained personnel and supportive care. A larger prospective study from multiple centres in India is required to conclusively state that the results of this study are truly representative of the country. In spite of these limitations, it is likely that this data is broadly representative of the experience of many tertiary centres in the country. Furthermore, this data is also likely to represent the challenges seen in many developing countries from where there is limited data.

The median age of 40 years (range: 1–79) in this cohort is strikingly different from that routinely reported in the literature from developed countries (Craig *et al*, 2012; Juliusson *et al*, 2012). This lower median age could be due to a combination tertiary centre referral bias and a different population pyramid structure in India and other developing economies, where the proportion of people over the age of 60 years is significantly lower. However, the possible role of additional genetic and environmental factors cannot be excluded.

From our data it is clear to us that the single most important factor for not proceeding with treatment was lack of financial resources (81%). A small proportion of patients that did not opt for treatment [*n* = 32 of 146 evaluated (22%); based on our follow‐up telephone interviews] went on to receive chemotherapy with curative intent from other centres after having been discharged from ours, but clearly the vast majority did not receive further treatment and succumbed to their disease. While patients in India frequently access alternative systems of medicine for treating various ailments, in this cohort, the number of patients who opted for alternative medicine was <2% of the population, based on the responses to the initial questionnaire (Table 2).

Fungal infections following induction chemotherapy are recognized to be associated with increased early mortality and to have an adverse impact on long‐term survival in patients with AML (Girmenia *et al*, 2014). The proportion of patients developing proven and probable IFI in AML following induction chemotherapy varies from 14% to 22% (Barreto *et al*, 2013; Gomes *et al*, 2013). In our series 28% of patients had evidence of possible or proven IFI. Four (14·8%) of the patients that died during induction had evidence of IFI.

A bigger challenge is the increased incidence of multidrug resistant (MDR) bacteria. This increased incidence is recognized as a global phenomenon (Johnson & Woodford, 2013). In the present study of 109 treated patients, 45 (42%) grew a GNB in one or more cultures post‐induction chemotherapy, 14 (31%) of whom had CRE growth on at least one blood culture. A CRE was isolated on blood culture in 7 (39%) of the patients that died during induction and an additional 5 of these induction deaths could be attributed to carbapenem‐resistant pseudomonas infection. The presence of MDR bacteria along with fungal infections adds significantly to the morbidity and mortality in AML. It also contributes significantly to the increased costs associated with treating AML. It is our experience that the major cost of treating patients in India is the cost of antibiotics and antifungal therapy. Recurrent cycles of neutropenia, duration of neutropenia and intensity of supportive care required (intensive care unit admission) are very closely linked to the overall cost of therapy. The current situation of MDR bacteria also limits the option of intensifying chemotherapy as a strategy to reduce the risk of relapse and improve long‐term survival.

At our centre we have been using arsenic trioxide (ATO)‐based therapy since 1998 for the treatment of acute promyelocytic leukaemia (APL; AML‐M3) (Mathews *et al*, 2006). It is interesting to note that prior to the introduction of ATO, the proportion of APL patients treated was similar to current proportions of AML (data not shown). With availability of generic ATO, the cost of therapy reduced to about 1/3rd to 1/4th of conventional therapy, predominantly as a result of the absence of neutropenia in the consolidation and maintenance arm of the protocol used (Mathews *et al*, 2006). A major advantage of this was that we could treat the majority of those newly diagnosed APL patients, who had similar constraints as described for AML patients in this study. However, with minimal financial assistance from non‐governmental organizations it is possible to treat almost every patient with newly diagnosed APL while the amount of financial support currently required for AML is beyond that which can be raised by similar means. During the period of this study, 47 patients were diagnosed with APL, all of whom received treatment at our centre. The clinical outcome of this cohort is best reflected in our previously published data (Mathews *et al*, 2010). Patients with a diagnosis of APL were not included in the present analysis because the treatment protocol is different and does not involve the same level of intensity as conventional AML therapy.

In conclusion, there are significant challenges in the management of AML in India. Induction deaths are related to a high incidence of MDR organisms and fungal infections. The biggest constraint is the cost of the treatment and the absence of a universal health security net to treat all patients with this diagnosis. Novel strategies, targets and targeted therapy which do not involve multiple cycles of prolonged cytopenia are urgently needed in the management of AML.

## Author contributions

CP: performed research, data accrual, analysed data and wrote the paper. BG: performed research, data accrual, fungal infection analysis and wrote the paper. AG: performed research, data accrual, analysed data. AK: performed research, data accrual, analysed data and financial analysis. PJ: performed research, data accrual, analysed data and financial analysis. AAA: performed research, immunophenotyping and analysed data. KML: performed research, biostatistician and analysed data. US: morphologist and laboratory data accrual. FNA: performed research and data accrual. AA: performed research and data accrual. AV: performed research and data accrual. VMS: performed research and cytogenetic analysis. AS: performed research and data accrual. PB: performed research, molecular analysis and analysed data. VM: designed study, performed research, data accrual, analysed data and wrote the paper.

## Conflict of interest disclosures

Nil.

## Supporting information


**Appendix S1.** Format of the questionnaire.
**Fig S1.** Distribution of time period in weeks of duration of symptoms prior to diagnosis at our centre.
**Table SI.** Comparison of baseline demographic characters, clinical features and laboratory parameters in newly diagnosed patients who received treatment and those that did not.
