# Supplementary material for: Acute myeloid leukaemia: challenges and real world data from India
Source: Br J Haematol. 2015 Apr 9;170(1):110–7. doi: 10.1111/bjh.13406 (PMC4864448; doi:10.1111/bjh.13406)
Supplement: Supplementary file 1 — Appendix S1. Format of the questionnaire. Fig S1. Distribution of time period in weeks of duration of symptoms prior to diagnosis at our centre. Table SI. Comparison of baseline demographic characters, clinical features and laboratory parameters in newly diagnosed patients who received treatment and those that did not. [file BJH-170-110-s001.docx]

**Acute myeloid leukaemia: Challenges and real world data from India**

Chepsy Philip^1^, Biju George^1^, Abhijeet Ganapule^1^, Anu Korula^1^, Punit Jain^1^, Ansu Abu Alex^1^, Kavitha M. Lakshmi^1^, Usha Sitaram^2^, Fouzia N. Abubacker^1^, Aby Abraham^1^, Auro Viswabandya^1^, Vivi M. Srivastava^3^, Alok Srivastava^1^, Poonkuzhali Balasubramanian^1^, Vikram Mathews^1^

1. Department of Haematology, Christian Medical College, Vellore, India.
2. Department of Transfusion Medicine and Immunohaematology, Christian Medical College, Vellore, India
3. Cytogenetics Unit, Christian Medical College, Vellore, India.

**Supplementary Information**

**Appendix 1:** Format of the questionnaire

**Demographics in Acute Myeloid Neoplasm (DAMN)**

**GENERAL DEMOGRAPHIC**

Name Patient ID/ UPN Sex: Year of birth:

State Country

Insured- Yes/No Company/other sponsored- yes/no

Distance from hospital (Approximately):

Birth order **1.**First **2.**Second **3.**Third **4.**Fourth **5.**Fifth or >

**SOCIO- ECONOMIC HISTORY:**

Type of work: Office worker Managerial/administrative Health worker Daily wages

Self-employed Agriculture/labourer

Equipment operator Factory worker

Home maker Student-school

Retired Other

If other; Details:

Residence: Owned /Rented Personal vehicle: - Yes / No

**PERSONAL & FAMILY HISTORY:**

Personal history: Tobacco: Yes/No Alcohol: Yes/No

Vegetarian: Yes/No

Previous cancer: Yes/No If Yes;

Treatment received: Surgery/ RT/ Chemotherapy/ Others

Siblings (number): Children (number):

Family history of cancer: If Yes; Details:

**TREATMENT HISTORY:**

Predominant symptoms (multiple ticks allowed)

a. Fever b. breathlessness c. Fatigability

d. Petechiae e. Bleeding f. Others g. If others: Details:

Duration of symptoms: in weeks

Prior Treatment after onset of symptoms: Antibiotics / ATT/ Antineoplastic agents / Steroids

(provide details)

**PERFORMANCE SCORE**

ECOG Definitions

0 Asymptomatic

1 Symptomatic, fully ambulatory

2 Symptomatic, in bed less than 50% of the day, but not bed ridden

3 Symptomatic, in bed more than 50% of the day but not bedridden

4 Bed ridden

**TREATMENT PLAN**

Conventional treatment- Yes/ No Curative/Palliative

Reasons for not proceeding with treatment

Religious Lack of social support

Economic Fear of side effects

Cultural Resignation

Alternative therapy Denial

If other; Details:

**Supplementary figure 1:** Distribution of time period in weeks of duration of symptoms prior to diagnosis at our centre.

Supplementary Table 1: Comparison of baseline demographic characters, clinical features and laboratory parameters in newly diagnosed patients who received treatment and those that did not.

| **Variable** | **Treated patients (n=109)**  **n(%) / Median (Range) /**  **Mean ±SD** | **Untreated patients (n=271)**  **n(%) / Median (Range) /**  **Mean ±SD** | **P value** |
| --- | --- | --- | --- |
| Age (years) | 38 (4- 68) | 41 (1-79) | 0.022 |
| Sex (male) | 73(67.0) | 171 (63.1) | 0.554 |
| Distance from hospital (km) | 500 (20-3000) | 616 (6-3200) | 0.005 |
| ECOG Score at diagnosis | n=109 | n=264 | - |
| 0-2 | 106 (97.2) | 254 (96.2) | 0.763 |
| 3-4 | 03 (2.8) | 10 (3.8) |  |
| Symptom duration (weeks) | 5.2 (±6.3) | 7.1(±7.1) | 0.000 |
| Age groups | - | - | - |
| ≤ 15 years | 23 (21) | 24 (8.9) | 0.001 |
| >15 - <60 years | 75 (68.8) | 196 (72.3) |  |
| ≥ 60 years | 11 (10) | 51 (18.8) |  |
| Haemoglobin (g/Lt) | 83 (36-151) | 74 (16-148) | 0.000 |
| WBC count (x10^9^/ Lt) | 13.3 (0.7-742.0) | 16.4 (0.2-920.0) | 0.837 |
| Platelet count (x10^9^/ L) | 46 (5.0-324.0) | 32 (2.0-394.0) | 0.013 |
| FLT3/NPM1 status | n=94 | n=49 | - |
| FLT3-/NPM1- | 67 (71.3) | 32 (65.3) | - |
| FLT3+/NPM1- | 3 (3.2) | 2 (4.1) | 0.096 |
| FLT3-/NPM1+ | 10 (10.6) | 12 (24.5) | - |
| FLT3+/NPM1+ | 14 (14.9) | 3 (6.1) | - |
| Cytogenetic Risk | n=108 | n=139 | - |
| Favourable | 13 (12.0) | 16 (11.5) | 0.717 |
| Intermediate | 73 (67.6) | 100 (71.9) |  |
| Adverse | 22 (20.4) | 23 (16.5) |  |
